# Twelve Brazilian families with X-linked Congenital Adrenal Hypoplasia: new rearrangements and new variants in the *NR0B1* gene

**DOI:** 10.1016/j.jped.2026.101538

**Published:** 2026-04-07

**Authors:** Adriana Mangue Esquiaveto-Aun, Sofia Helena Valente de Lemos-Marini, Mara Sanches Guaragna, Taís Nitsch Mazzola, Karina de Ferran, Renata Szundy Berardo, Daniel Luis Schueftan Gilban, Gabrielle Sormanti Schnaider, Cristiane Kopacek, Isabel Rey Madeira, Marilza Leal Nascimento, Maricilda Palandi de Mello, Gil Guerra-Junior

**Affiliations:** a Universidade Estadual de Campinas (UNICAMP), Faculdade de Ciências Médicas (FCM), Programa de Pós-Graduação em Saúde da Criança e do Adolescente, Campinas, SP, Brazil; b Universidade Estadual de Campinas (UNICAMP), Centro de Biologia Molecular e Engenharia Genética, Laboratório de Genética Humana, Campinas, SP, Brazil; c Universidade Estadual de Campinas (UNICAMP), Faculdade de Ciências Médicas (FCM), Departamento de Pediatria, Campinas, SP, Brazil; d Universidade Estadual de Campinas (UNICAMP), Faculdade de Ciências Médicas (FCM), Centro de Investigação em Pediatria (CIPED), Campinas, SP, Brazil; e Universidade Federal do Rio de Janeiro (UFRJ), Instituto de Puericultura e Pediatria Martagão Gesteira, Unidade de Endocrinologia e Metabolismo, Rio de Janeiro, RJ, Brazil; f Hospital Federal dos Servidores do Estado, Departamento de Pediatria, Rio de Janeiro, RJ, Brazil; g Universidade do Estado do Rio de Janeiro (UERJ), Faculdade de Ciências Médicas (FCM), Departamento de Pediatria, Rio de Janeiro, RJ, Brazil; h Universidade do Vale do Sapucaí (UNIVAS), Faculdade de Ciências Médicas (FCM), Departamento de Pediatria, Pouso Alegre, MG, Brazil; i Universidade Federal do Rio Grande do Sul (UFRGS), Faculdade de Medicina, Departamento de Pediatria, Porto Alegre, RS, Brazil; j Universidade Federal de Ciências da Saúde de Porto Alegre (UFCSPA), Programa de Pós-Graduação em Pediatria, Porto Alegre, RS, Brazil; k Universidade Federal de Santa Catarina (UFSC), Faculdade de Medicina, Departamento de Pediatria, Florianópolis, SC, Brazil

**Keywords:** *NR0B1* gene, X-hypo AC, Primary adrenal insufficiency

## Abstract

**Objective:**

To characterize the molecular spectrum of NR0B1 variants associated with X-linked congenital adrenal hypoplasia (X-hypoAC) in a multicenter Brazilian cohort and to highlight the clinical implications of early molecular diagnosis.

**Methods:**

The authors investigated 12 unrelated families referred to pediatric endocrinology centers across Brazil with a clinical diagnosis of early-onset adrenal insufficiency. Clinical data, biochemical profiles, and follow-up information were collected. Genetic testing of the NR0B1 gene was performed by Sanger sequencing to detect single-nucleotide and small insertion/deletion variants, complemented by MLPA to identify large deletions or rearrangements. Variants were classified according to the American College of Medical Genetics and Genomics (ACMG) guidelines.

**Results:**

All probands presented with adrenal insufficiency within the first months or years of life, often requiring hospitalization and lifelong glucocorticoid and mineralocorticoid replacement. Molecular analysis revealed a heterogeneous spectrum of NR0B1 pathogenic variants, including frameshift, nonsense, and missense variants, as well as large and small deletions, which together accounted for nearly half of the cases. Clinical follow-up confirmed that, in addition to adrenal insufficiency, affected individuals frequently developed hypogonadotropic hypogonadism during puberty, with infertility documented in adulthood. The identification of novel variants contributes to expanding the mutational spectrum of NR0B1and reinforces the genotype–phenotype variability in X-hypoAC.

**Conclusions:**

This study represents one of the largest Brazilian series of X-hypoAC. The present findings broaden the molecular landscape of NR0B1 variants and underscore the relevance of early genetic testing to differentiate X-hypoAC from congenital adrenal hyperplasia, optimize long-term clinical management, and provide accurate genetic counseling for affected families.

## Introduction

X-linked congenital adrenal hypoplasia (X-hypoAC) (OMIM #300,200) is a rare and potentially fatal condition caused by variants in the *NR0B1* gene (OMIM *300,473). X-hypoAC presents clinically as early-onset primary adrenal insufficiency (PAI), similar to congenital adrenal hyperplasia due to 21-hydroxylase deficiency, but commonly progresses to hypogonadotropic hypogonadism at puberty and infertility in adulthood [1].

The *NR0B1* gene, previously called *DAX1*, is located on Xp21.3 and encodes an orphan nuclear receptor that acts as a coregulator, inhibiting the transcriptional activity of other nuclear receptors [2]. It is mainly expressed in the adrenal, gonadal, anterior pituitary and hypothalamus tissue, being responsible for both embryonic development and maintenance of these organs [3].

The presence of pathogenic variants in the *NR0B1* gene as a cause of X-hypoAC was first described by Zanaria et al. [4] when studying individuals who had adrenal hypoplasia with hypogonadotropic hypogonadism, associated with Duchenne muscular dystrophy and glycerol kinase deficiency. Genetically, these individuals had a deletion in the short arm of the X chromosome (contiguous gene syndrome - OMIM #300,679) that included the deletion of the DMD, GK and NR0B1 genes, respectively. Currently, >250 pathogenic variants have been described throughout the NR0B1 gene (https://www.hgmd.cf.ac.uk/ac/gene.php?gene=NR0B1 – accessed on 02/26/2025), half of them characterized by small and large deletions, with no hot-spot regions or well-established genotype-phenotype relationship [5]. The objective of this report is to describe different pathogenic variants in the *NR0B1* gene as the cause of PAI in boys from different Pediatric Endocrinology centers in Brazil, after excluding the diagnosis of congenital adrenal hyperplasia.

## Methods

This study was conducted at the Center for Molecular Biology and Genetic Engineering (CBMEG) of the State University of Campinas (UNICAMP) and included patients followed at the UNICAMP Hospital and other university hospitals located in the southeast and south of Brazil.

Patients who had received a clinical diagnosis of PAI were included, most of them were characterized by the presence of vomiting with severe dehydration, weight loss, hypoglycemia accompanied or not by seizures, skin hyperpigmentation, cardiorespiratory arrest; followed by laboratory diagnosis of hyponatremia, hyperkalemia, hypoglycemia, hypocortisolism and increased plasma ACTH concentration (Table 1).

**Table 1:** Clinical, laboratory and molecular findings of 12 families (13 cases) of Primary Adrenal Insufficiency due to alteration in the NR0B1 gene.

Families	Age of onset of symptoms	Initial presentation	Treatment	Initial ACTH (RV: < 46 pg/ml)	Initial cortisol (RV: 5–25 µg%)	Sodium (RV: 139–146 meq/l)	Potassium (RV: 3.4–4.7 meq/l)	Additional findings	Molecular diagnosis	ACMG classification
1	2 weeks	Vomiting, severe dehydration	GC e MC	220	< 0.1	124	5.6	Hypogonadotropic hypogonadism	Deletion–insertion–inversion–deletion complex rearrangement in *NR0B1* gene	Pathogenic (criteria 1A e 2A)
2	4 days (age of diagnosis: 3 years)	Frequent episodes of dehydration	GC e MC	4,17	5.5	126	3.8	Duchenne muscular dystrophy Hypertriglyceridemia	Deletion genes: *NR0B1, DMD and GK* (Contiguous gene syndrome) NR0B1: chrx: 30,304,206–30,309,390; DMD: chrx: 31,119,222–33,211,549; GK: chrx: 30,653,359–30,731,462	Pathogenic (criteria 1A e 2A)
3	3 weeks	Hypoglycemia, vomiting, dehydration	GC e MC	931	12.0	119	7.3	-	Deletion of the entire gene *NR0B1* (chrx 30.082.120 - 30.087.136)	Pathogenic(criteria 1A e 2A)
4	1 month	Low weight gain, vomiting, dehydration	GC e MC	681	0.8	98	5.6	-	NM_000475.5:c1292del p.Ser431Ilefs*6	Probably pathogenic (PVS1_*S* + PM2+PP4)
5	15 days	Dehydration, hypoglycemia, hyponatremia	GC e MC	NA	NA	NA	NA	Duchenne muscular dystrophy Hypertriglyceridemia	Deletion genes: *NR0B1, DMD and GK* (Contiguous gene syndrome) NR0B1: chrx: 30,304,206–30,309,390; DMD: chrx: 31,119,222–33,211,549; GK: chrx: 30,653,359–30,731,462	Pathogenic (criteria 1A e 2A)
6 (case 1)	3 months	Vomiting, weight loss, dehydration	GC e MC	NA	NA	118	6.0	Brother died at 37 days old due to vomiting and dehydration	Deletion of the entire gene *NR0B1* (chrx 30.082.120 - 30.087.136)	Pathogenic (criteria 1A e 2A)
6 (case 2)	2 weeks	Vomiting, dehydration	GC e MC	196	7.3	127	8.8	Siblings: 1) died at 37 days of life due to vomiting and dehydration; 2) PAI	Deletion of the entire gene *NR0B1* (chrx 30.082.120 - 30.087.136)	Pathogenic (criteria 1A e 2A)
7	1 week	Skin hyperpigmentation, weight loss, hypoglycemia	GC e MC	1,25	< 0.1	126	6.4	-	Deletion of the entire gene *NR0B1* (chrx 30.082.120 - 30.087.136)	Pathogenic (criteria 1A e 2A)
8	1 week	Loss of appetite, weight loss, vomiting, dehydration (2x cardiorespiratory arrest)	GC e MC	171 (in treatment)	6.7	NA	NA	-	NM_000475.5:c.497G>*A* (p.Arg166Gln)	VUS (PM2 + BS2)
9	17 days	Severe dehydration	GC e MC	NA	NA	117	8.0	-	NM_000475.5:c.1144A>*T* (p.Lys382*)	Pathogenic (PVS1+PM 2+PP4)
10	1 month	Vomiting, low weight gain, dehydration	GC e MC (treated for up to 3–4 years - was suspended for 1 year and then returned to use)	1,25	NA	96	NA	Hypogonadotropic hypogonadism	NM_000475.5:c.74_86 del (p.Ala25Valfs*56)	Pathogenic (PVS1+PM2+PP4)
11	3 days	Abdominal distension (3rd DL), hypoactivity, dehydration (11th DL), cardiorespira-tory arrest (13th DL)	GC e MC	1567	1.4	111	7.5	Older (deceased) and younger brother with adrenal insufficiency	NM_000475.5:c264del (p.Lys88Asnfs*176)	Pathogenic (PVS1+PM2+PP4)
12	2 years	Severe hypoglycemia (seizure)	GC e MC	6214	< 0.1	116	4.9	Neuropsycho-motor developmental delay/ alacrimia	NM_000475.5: c.970dup (p.Arg324Lysfs*65)	Probably Pathogenic PVS1_*S*+PM2+PP4

DL, days of life; GC, glucocorticoid; MC, mineralocorticoid; ACTH, adrenocorticotropic hormone; RV, reference value; NA, not available; PAI, primary adrenal insufficiency.

A consent form was obtained from all guardians of the individuals participating in the study, previously approved by the Ethics Committee (CAAE 29,796,420.8.0000.5404). Cases of congenital adrenal hyperplasia diagnosed in the laboratory due to the accumulation of adrenal precursors were excluded.

Each *NR0B1* exon and its flanking regions were amplified by PCR from genomic DNA [6] using primers designed by the free GeneRunner v3.1 software (https://gene-runner.software.informer.com/6.0). The amplicons produced by each primer combinations were purified on 1% agarose gel electrophoresis with the Wizard SV Gel & PCR clean-up system (Promega, Madison, WI, USA), and both sense and antisense strands were sequenced using the ABI PRISM Big Dye Terminator v3.1 Cycle Sequencing Kit (ABI PRISM/PE; Applied Bio-systems, Foster City, CA, USA) and analyzed by the ABI 3500xL sequencer (Applied Biosystems) using the same primers as in the PCR. Sequencing data were analyzed using the free software 4peaks v.1.7.1 (https://nucleobytes.com/4peaks/index.html) and the sequences aligned with the reference sequence of the NR0B1 gene (ENSG00000169297, www.ensembl.org) by the free software CLC v.8 (Qiagen, Hilden, Germany), for comparison. In the absence of amplification after the PCR reaction, the MLPA technique (SALSA MLPA P185 Intersex version C1 or C2 - MRC—Holland, Amsterdam, Netherlands) was used to verify deletion in the entire gene or part of it. The analysis of the obtained fragments was performed on the ABI 310 sequencer (ABI PRISM/PE Biosystems, Foster City, CA, USA), and the results were analyzed using the Genescan and Genotyper software (Applied Biosystems, Foster City, CA, USA). The pathogenicity of sequence variants and deletions of one or more genes was classified by the criteria of the American College of Medical Genetics and Genomics (ACMG).

## Results

The study included 13 boys from 12 unrelated families whose suspected diagnosis of primary adrenal insufficiency (PAI) occurred predominantly within the first month of life. Clinical, laboratory, and molecular findings, and additional relevant information are summarized in Table 1.

From a clinical standpoint, recurrent vomiting associated with severe dehydration was a universal presenting feature, frequently accompanied by poor weight gain and hypoglycemia. Cases 8 and 11 progressed to cardiorespiratory arrest, underscoring the severity of the clinical presentation. Cutaneous hyperpigmentation was documented in case [7].

Biochemically, hyponatremia and hyperkalemia were observed in all patients for whom laboratory data were available (some cases were referred for molecular investigation without complete biochemical records). Most patients exhibited elevated plasma ACTH levels and reduced morning serum cortisol concentrations. However, a subset had normal hormonal measurements, either due to technical limitations of laboratory assays or prior initiation of glucocorticoid replacement therapy.

Additional clinically relevant findings were noted. Two patients had a family history of early sibling deaths of unknown cause (families 6 and 11). Delayed puberty with subsequent diagnosis of hypogonadotropic hypogonadism developed during follow-up in two cases (families 1 and 10). Two patients (families 2 and 5) were also diagnosed with Duchenne muscular dystrophy and hypertriglyceridemia, raising suspicion for Xp21 contiguous gene deletion syndrome. In family 12, a history of recurrent severe hypoglycemic seizures beginning at two years of age, associated with neuropsychomotor developmental delay and alacrimia, initially suggested Allgrove syndrome (triple A syndrome; OMIM #231,550). This diagnosis was excluded after sequencing confirmed the absence of pathogenic variants in the AAAS gene. All patients were treated with both glucocorticoid and mineralocorticoid replacement therapy.

## Discussion

The diagnosis of X-hypoAC should be considered in all boys who present with an adrenal insufficiency crisis in the first months of life or in early childhood, especially in those with a family history of early death, after congenital adrenal hyperplasia has been ruled out. Clinical diagnosis is still challenging, since the signs and symptoms of an adrenal crisis can easily be confused with other conditions, delaying the institution of treatment and increasing the risk of death [8].

**Figure 1:**
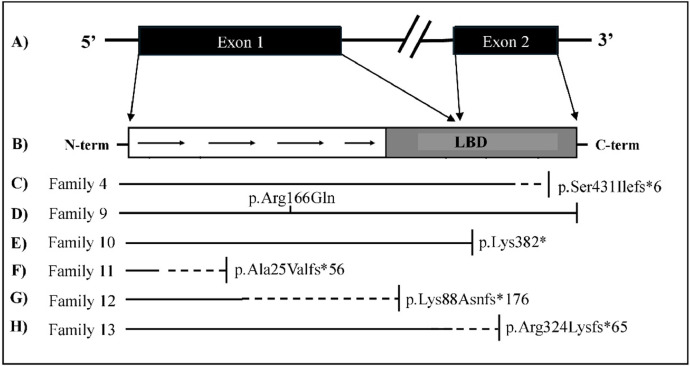
Schematic representation of the *NR0B1* gene and the variants identified in the patients studied. A. Schematic of the NR0B1 gene: exon 1 encoding the DNA-binding domain (DBD) in the amino-terminal portion (N-term) and part of the ligand-binding domain (LBD) and exon 2 encoding the final portion of the LDB in the carboxy-terminal portion (C-term). Below are represented: B) Receptor domains. C*) Schematic representation of the consequence of the frameshift variant found in family 4. D) Missense variant found in family 8. E) Nonsense variant found in family 9, with the stop codon represented by the vertical line. F, G and H*) Frameshift variants found 10, 11 and 12. * The solid line represents the intact protein, the dashed line represents the truncated region, with the premature stop codon represented by the vertical line.

Pathogenic variants in the *NR0B1* gene are found in approximately 40% of cases of PAI in children [9]. All cases in the series of adrenal insufficiency patients were molecularly diagnosed with defects in the *NR0B1* gene, after ruling out congenital adrenal hyperplasia, including in the case of family 12, with clinical suspicion of Allgrove syndrome. Two points are worth highlighting: 1) The variants described here were located throughout the gene (Figure 1), reinforcing the idea that there is no predisposition region for the occurrence of variants in the *NR0B1* gene; 2) There is no well-established genotype-phenotype relationship, since variants with a location similar to those reported here as causing early-onset X-hypoAC have been described in patients with adult-onset X-hypoAC (late-onset form) [10].

The molecular diagnosis is important for both genetic counseling and treatment; in addition, it offers the possibility of detecting affected family members before the onset of symptoms, avoiding more serious clinical outcomes.

## Funding

This work was supported by grants from Conselho Nacional de Desenvolvimento Científico e Tecnológico (CNPq # 471121/2004–5) and Fundação de Amparo à Pesquisa do Estado de São Paulo (FAPESP # 2008/54776–1).

## Data availability

The data that support the findings of this case report are available from the corresponding author on request (adriana.aun@slmandic.edu.br).

## Conflicts of interest

The authors declare no conflicts of interest.
